# Mutational processes contributing to the development of multiple myeloma

**DOI:** 10.1038/s41408-019-0221-9

**Published:** 2019-08-06

**Authors:** Phuc H. Hoang, Alex J. Cornish, Sara E. Dobbins, Martin Kaiser, Richard S. Houlston

**Affiliations:** 1 Division of Genetics and Epidemiology, The Institute of Cancer Research, London, UK; 2 Division of Molecular Pathology, The Institute of Cancer Research, London, UK

**Keywords:** Cancer genetics, Cancer genomics

## Abstract

To gain insight into multiple myeloma (MM) tumorigenesis, we analyzed the mutational signatures in 874 whole-exome and 850 whole-genome data from the CoMMpass Study. We identified that coding and non-coding regions are differentially dominated by distinct single-nucleotide variant (SNV) mutational signatures, as well as five de novo structural rearrangement signatures. Mutational signatures reflective of different principle mutational processes—aging, defective DNA repair, and apolipoprotein B editing complex (APOBEC)/activation-induced deaminase activity—characterize MM. These mutational signatures show evidence of subgroup specificity—APOBEC-attributed signatures associated with *MAF* translocation t(14;16) and t(14;20) MM; potentially DNA repair deficiency with t(11;14) and t(4;14); and aging with hyperdiploidy. Mutational signatures beyond that associated with APOBEC are independent of established prognostic markers and appear to have relevance to predicting high-risk MM.

## Introduction

Cancers have variable numbers of somatic mutations that have accumulated during the life history of the tumors as a consequence of diverse cellular processes, including defective DNA replication or DNA repair, and exposure to endogenous or exogenous DNA-damaging agents^1,2^. Each of these processes results in mutational signatures, which serve as proxy for the cellular processes that have gone amiss. Mathematical deconvolution^3^ of these mutational signatures in large pan-cancer series has revealed multiple distinct signatures^1^, several of which are associated with known etiologies, but many remain unexplained^1,4,5^. Hence, studying the mutational signatures of cancers provides a mechanism for gaining insight into the etiological basis of tumor development.

Multiple myeloma (MM) is an incurable malignancy of plasma cells whose pathogenesis is only partially understood^6^. Approximately 40% of MM tumors harbor chromosome translocations leading to over-expression of oncogenes (including *CCND1*, *CCND3*, *MAF*, *MAFB*, *WHSC1*/*MMSET*, and *FGFR3*) through juxtaposition to the immunoglobulin heavy-chain locus^6^. Other tumors exhibit hyperdiploidy (HD), which is also considered to be an important initiating event^6^. Whole-exome sequencing (WES) and whole-genome sequencing (WGS) studies have so far identified over 40 driver genes that are recurrently altered in MM^6–10^. However, the molecular mechanisms giving rise to these mutations are yet to be fully elucidated.

Here we report a comprehensive analysis of the mutation signatures of over 800 MM genomes. We identify major mutational signatures in MM reflective of three known principle mutational processes: aging^1,11,12^, DNA repair deficiency^1,12–17^, and activation-induced deaminase (AID)/apolipoprotein B editing complex (APOBEC) activity (signature 2, 9, and 13)^1,13,18,19^. These mutational signatures show subgroup specificity and are reflective of the molecular mechanisms involved in tumorigenesis. Additionally, we show that information on mutational signatures beyond that associated with APOBEC has relevance to predicting patient prognosis and defining high-risk MM.

## Results

### Genome sequencing of multiple myeloma

To examine the diversity of mutational signatures, we analyzed overlapping WGS and WES data on 850 and 874 MM tumor-normal pairs, respectively, generated by the Relating Clinical Outcomes in Multiple Myeloma to Personal Assessment of Genetic Profile Study (CoMMpass, IA10 release). The frequency of the MM major subgroups—HD, t(11;14), t(4;14), t(14;16), t(14;20), and t(8;14) *MYC* translocation—is similar to other unselected series of patients who have been reported^6^ (Supplementary Table 1). We used the high-coverage WES data (120–150×, 136,074 single-nucleotide variants [SNVs]) to analyze coding regions and the low-coverage WGS data (6–12×, 1,348,881 SNVs and 44,155 structural variants [SVs]) to provide genome-wide insights into clonal mutations associated with early processes underlying tumorigenesis^7,20^.

### Mutational signatures in multiple myeloma

**Fig. 1:**
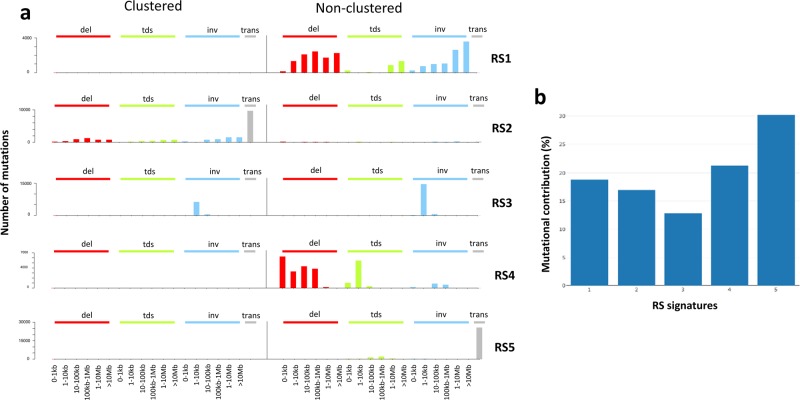
De novo structural rearrangements signatures. **a** Five de novo structural rearrangement signatures (RSs) extracted in multiple myeloma. **b** Cumulative mutational contribution of the structural rearrangements across 850 whole-genome sequencing (WGS) samples. Del: deletions; tds: tandem duplications; inv: inversions; trans: translocations

Application of non-negative matrix factorization (NMF)^11^ (Supplementary Fig. 1) to extract de novo SNV mutational signatures did not identify any novel mutational signatures (Supplementary Figs. 2 and 3), consistent with a recent analysis on CoMMpass exome dataset^21^. Overall, a total 9 of the 30 mutational signatures referenced by COSMIC (Catalog of Somatic Mutations in Cancer) were seen at >1% mutational contribution in the WGS data (Supplementary Table 2); signature 1 related to aging^1^; 2 and 13 to activity of the APOBEC family of cytidine deaminases; 9 to polymerase η implicated with the activity of AID during somatic hypermutation^1,18,19^; signature 30 reflective of mismatch repair deficiency^17^; and signature 16 which has as yet an unknown etiology. We also extracted flat signatures 3, 5, and 8 in tumors - indicative of DNA repair deficiency (homolgous recombination deficiency and nucleotide repair deficiency)^1^^,^^12–16^. In view of the potential ambiguous assignment of these three signatures^22,23^, we considered them collectively thereafter. We did however identify five novel de novo structural rearrangement signatures (RSs) (Fig. 1): RS1 (19% of SVs across samples)—characterized by non-clustered deletions, large-scale tandem duplications and inversions; RS2 (17%)—characterized by clustered translocations; RS3 (13%)—characterized by inversions; RS4 (21%)—characterized by non-clustered small-scale deletions and tandem duplications; RS5 (30%)—characterized by non-clustered translocations. We therefore focused on the nine major SNV and five de novo SV mutational signatures for subsequent analyses.

Following on from this, we examined the contributions of the nine major COSMIC SNV mutational signatures in both WES and WGS datasets. The signature profiles recovered from the analysis of clonal WES and exome-restricted WGS data were highly correlated (*r* = 1.00, Spearman’s correlation, Supplementary Fig. 4, Supplementary Table 3). Hence, while the average sensitivity to detect clonal SNVs from the WGS data is 20–35%^7^, these findings indicate that the mutational signatures identified by WGS are valid and representative of early mutational processes in MM. We also observed a high concordance of mutational signature in WES data from CoMMpass and that reported by Walker et al.^8^ (*r* = 0.86, Spearman’s correlation, Supplementary Fig. 5, Supplementary Table 4), reflecting the generalizability of our observations. No significant association between the major COSMIC SNV signatures and those associated with rearrangements was seen (Supplementary Table 5).

### Influence of DNA replication and transcription on mutational signatures

**Fig. 2:**
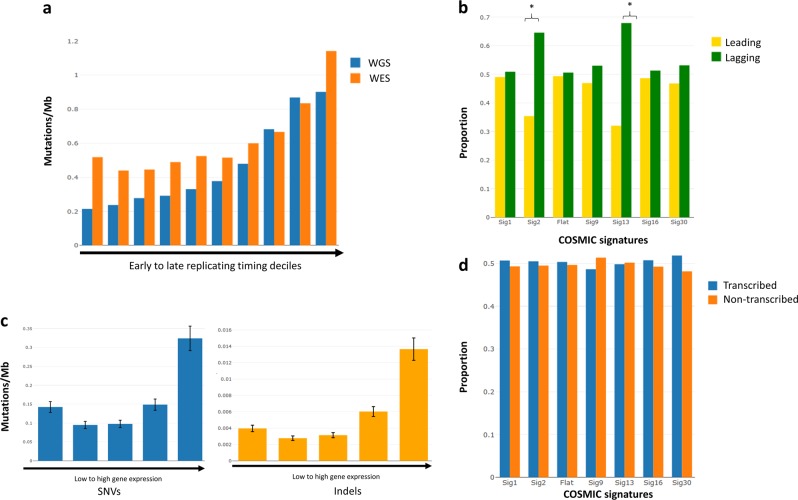
Relationship between replication and transcription in mutational processes. **a** Mutation rates across different DNA replication timing bins for single-nucleotide variants (SNVs). Whole-genome sequencing (WGS) mutation rate (blue) was estimated from low-coverage WGS data (6–12×). Whole-exome sequencing (WES) mutation rate (orange) was estimated from high-coverage WES data (120–150×) with variants called by at least two variant callers. **b** Proportion of mutations on leading and lagging strands per signature based on WGS data. Asterisks indicate significant asymmetry (*Q* < 0.05 and strand imbalances >30%). **c** Relationship between transcriptional level and mutation rate. The range of number of genes across all samples included in each FPKM category (from low to high gene expression) are category 1: 4062–6800 (median 4209); category 2: 1323–4062 (median 3914); category 3: 4060–4062 (median 4061); category 4: 4060–4061 (median 4061); category 5: 4062. Error bars represent the 95% confidence intervals. **d** Proportion of mutations on transcribed and non-transcribed strands across major signatures based on WES data. WGS: whole-genome sequencing; WES: whole-exome sequencing; SNVs: single-nucleotide variants; FPKM: fragments per kilobase of exons per million reads. Flat signatures include COSMIC signatures 3, 5, and 8

The impact of DNA replication and transcription on mutational signatures was broadly consistent with observations previously made in the analyses of other cancers^11,12,24^. Specifically, an overall increased mutation rate in late-replicating regions was shown (*P* < 1.0 × 10^−4^) (Fig. 2a, Supplementary Table 6), with the exception of signature 13 having higher mutation rate in early-replicating regions (*P* < 1.0 × 10^−4^, Supplementary Fig. 6, Supplementary Table 7), consistent with generalized replication time-dependent DNA damage mechanisms that operate in other cancers such as those of the breast^12^ and liver^11^. The difference in how replication timing influences mutation rates in signatures 2 and 13, which are both associated with APOBEC activity, suggests an intrinsically different mutational processes linked to DNA replication consistent with the model previously described^12^.

Similarly, as previously documented, strong replicative strand asymmetry (>30% imbalances)^12^ was shown with respect to signatures 2 (*Q* *=* 4.0 × 10^−16^) and 13 (*Q* = 4.0 × 10^−16^) with higher mutation in the lagging strand (Fig. 2b, Supplementary Table 8). These findings are consistent with APOBEC activity primarily affecting lagging strands.

Overall, increased mutation rate was associated with increased transcription, suggesting the mutagenic role of the transcriptional process in MM (Fig. 2c). This contrasts markedly to hepatocellular carcinoma^11^, suggesting that transcription-associated mutagenesis may overwhelm transcription-coupled repair in MM^25^. Moreover, strikingly elevated mutation rates of both SNVs and indels were shown for highly expressed genes (Fig. 2c). A number of these highly expressed genes (i.e., FPKM (fragments per kilobase of exons per million reads) >100), which are also frequently mutated, including *EGR1*^26^, *XBP1*^27^, *BTG2*^28^, *DDX5*^29^, and *NFKBIA*^9^ (Supplementary Table 9), have well-established roles in plasma cell differentiation and MM. The strong replicative, but weak transcriptional mutational asymmetry (Fig. 2d, Supplementary Table 10) seen in MM is consistent with the mutual exclusivity trend of replicative and transcriptional asymmetries shown in many cancers^24^.

### Mutational signatures in coding and non-coding regions

**Fig. 3:**
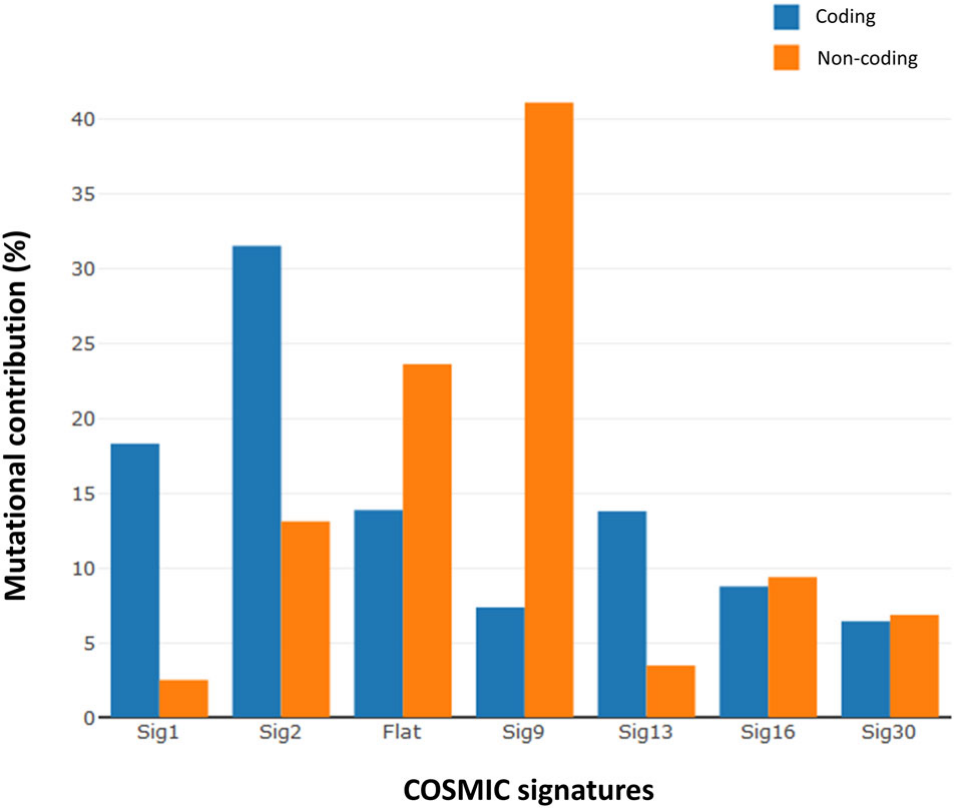
Contribution of each single-nucleotide variant mutational signature in coding (blue) and non-coding (orange) regions. Flat signatures include COSMIC signatures 3, 5, and 8

A significant difference in all mutational signatures within coding and non-coding regions was shown (Fig. 3, Supplementary Table 11), implying different genomic regions are subject to specific mutational processes, consistent with earlier observations^30^. AID-attributed signature 9 predominates in non-coding regions, whereas exonic mutations are dominated by signatures 1, 2, and 13, implicating aging and APOBEC signatures as important.

### Relationship between mutational signatures and kataegis

Local hypermutated regions of tumor genomes, or kataegis, have been observed in MM^9,31^ and other B cell malignancies^1^. We examined COSMIC mutational signatures contributing to kataegis (defined on the basis of average inter-mutation distance ≤1 kb;^3,32^ Supplementary Table 12a), which were detected in 9% of samples (71/874). We did not observe significant and consistent enrichment of COSMIC signatures at kataegis foci compared to other mutations in tumors with and without kataegis detected (Supplementary Table 12b). We identified 70 genes disrupted by kataegis (Supplementary Table 13), including *CCND1*, *CCND3*, *MAF*, and *FZD2*, which are often affected by chromosomal rearrangements^6,33^. Globally, 62% of kataegis foci co-localize with 5% of somatic structural arrangement sites (Supplementary Fig. 7), consistent with previous finding that most genomic rearrangements do not feature kataegis in nearby regions^1^.

### Mutational signatures and myeloma subgroups

We observed significant association between specific mutational signatures and MM subgroups (Table 1). Signature 1 was enriched in HD MM (*Q* = 3.2 × 10^−4^) consistent with the correlation between age and frequency of HD^34^ (Supplementary Table 14). APOBEC-attributed signatures 2 and 13 were enriched in *MAF*-translocation subgroups—t(14;16) (*Q* = 1.7 × 10^−15^ and *Q* = 3.5 × 10^−19^, respectively), t(14;20) (*Q* = 1.4 × 10^−3^ and *Q* = 6.4 × 10^−6^, respectively)—and to a lesser extent in t(4;14) (only signature 2, *Q* = 9.3 × 10^−6^) consistent with previous reports^7,35^. Flat COSMIC signatures, attributable to DNA repair deficiency, were enriched in t(11;14) MM (*Q* = 3.3 × 10^−4^) and t(4;14) MM (*Q* = 0.033). We observed an enrichment of non-clustered deletions, large-scale tandem duplications, and inversions RS1 (*Q* = 3.8 × 10^−6^); and clustered translocation RS2 (*Q* *=* 0.010) signatures in t(4;14) MM (Supplementary Table 15). Although speculative it is possible that the t(4;14) translocation, which leads to up-regulation of histone methyltransferase (MMSET), may affect genomic instability through some as yet undisclosed epigenetic mechanism.

**Table 1:** Association of major myeloma subgroups and mutational signature (*Q* < 0.05)

Subgroup	Signature enrichment	Suggested etiologies
Hyperdiploidy	Signature 1	Aging
t(11;14)	Flat signatures	Potentially DNA repair deficiency
t(4;14)	Signatures 2, 30, and flat signatures	APOBEC and potentially DNA repair deficiency
t(14;16)	Signatures 2 and 13	APOBEC
t(14;20)	Signatures 2 and 13	APOBEC
*MYC*	NA	NA

*MYC* t(8;14) *MYC*-translocation subgroup, *APOBEC* apolipoprotein B editing complex, *NA* not available

We further explored the links between established prognostic mutational events (1p deletion, 1q gain, 17p deletion, and *TP53* mutations) with mutational signatures (Supplementary Table 16). Associations between chromosome-arm events at 1p and 1q with COSMIC signatures 2, 13, and RS1 (*Q* < 0.05) and between *TP53* mutations tumors with RS1 (*Q* = 0.033) and RS2 (*Q* = 7.4 × 10^−3^) raise the possibility of causal relationships.

### Mutational signatures and driver genes

**Fig. 4:**
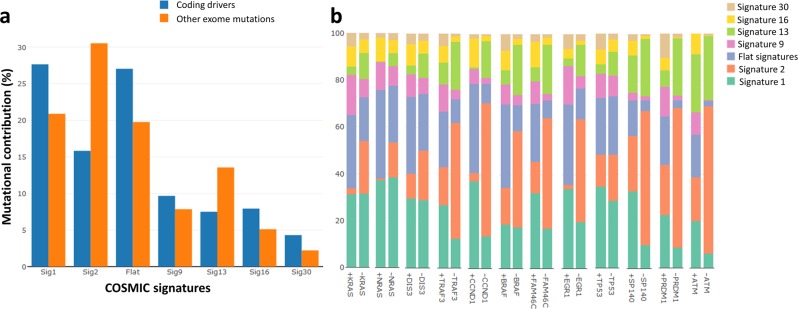
Mutational signatures associated with driver genes. **a** Cumulative mutational contribution of mutational signatures across 50 multiple myeloma (MM) driver genes^7–10^ (blue, 1679 mutations in total) and other exonic mutations (orange). **b** Normalized cumulative mutational contribution of signatures with top ten contribution for most frequently mutated MM driver genes (+) vs. other mutations (−) in tumors with the corresponding driver gene being mutated: *KRAS* (*n* = 247), *NRAS* (*n* = 204), *DIS3* (*n* = 104), *TRAF3* (*n* = 83), *CCND1* (*n* = 78), *BRAF* (*n* = 70), *FAM46C* (*n* = 70), *EGR1* (*n* = 65), *TP53* (*n* = 52), *SP140* (*n* = 30), *PRDM1* (*n* = 26), and *ATM* (*n* = 19); *n*: number of mutations. Flat signatures include COSMIC signatures 3, 5, and 8

To identify etiological mutational processes underlying driver mutations in MM, we compared mutational contribution in driver genes to other exonic mutations. Overall, the same diversity of processes in driver mutations was seen as in other coding mutations, but with differences: lower contribution of signatures 2 and 13; and higher contribution of signatures 1, 9, 16, 30, and the flat signatures in coding regions of driver genes, compared to other exonic mutations (Fig. 4, Supplementary Table 17). Notably, we observed an over-representation of signatures reflective of aging with *CCND1* and *DNAH5* mutations, and AID with *EGR1* mutations (Fig. 4, Supplementary Table 18). In contrast, a relative under-representation of signatures 2 and 13 suggests that APOBEC mutations are ubiquitous mutational processes and they do not specifically affect driver genes. Driver genes were replicated earlier than other coding genes (*P* < 2.2 × 10^−16^, Wilcoxon’s rank-sum test) and we therefore assessed whether this difference could explain enrichment of the signatures. APOBEC signature 2 is enriched in late-replicating regions (Supplementary Fig. 6, Supplementary Table 7); hence, the tendency of driver genes to be replicated early may explain the lower frequency of signature 2 mutations associated with driver genes. Signatures 1, 9, 16, 30, and the flat signatures were also associated with late-replicating regions (Supplementary Fig. 6, Supplementary Table 7), but conversely were more frequently associated with driver gene mutations. To test if the enrichment of mutational processes in driver genes were due to positive selection of certain mutations, we excluded all mutations that occurred at the exact same position in multiple tumors (46% of mutations) and repeated the analysis. Exclusion of recurrent mutations did not change the overall results, inferring that positive selection of specific mutations did not bias the analysis. We did not observe any significant transcriptional strand bias across mutational signatures (Fig. 2d), suggesting that the differences in mutational contribution between driver genes and other exonic mutations are unlikely to be influenced by transcription.

### Prognostic impact of mutational signatures

**Fig. 5:**
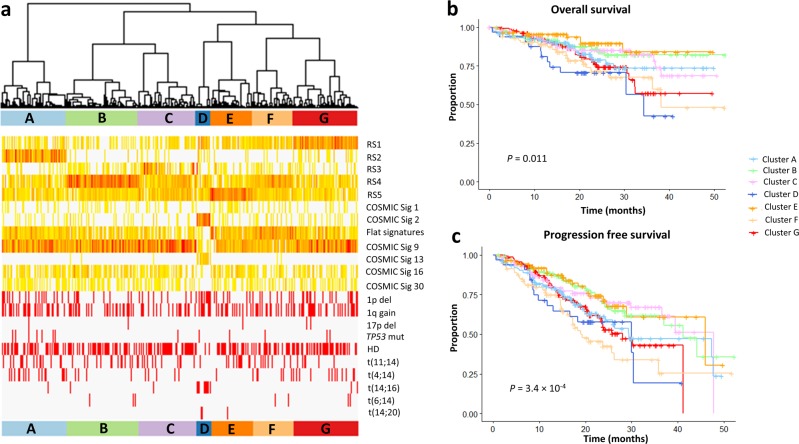
Integrative clusters based on mutational signatures and patient prognosis. **a** Heatmap showing proportions of rearrangement signatures and major COSMIC (Catalog of Somatic Mutations in Cancer) signatures in unsupervised hierarchical clusters. Flat signatures include COSMIC signatures 3, 5, and 8. The lower panel shows distribution of translocations, prognostic chromosome-arm events, and *TP53* non-synonymous mutations across all samples. **b** Progression-free survival and **c** overall survival across different cluster groups. The global *P* values across all cluster groups were calculated to assess whether there is survival difference between groups

We next investigated the prognostic impact of mutational signatures using the prospective data from CoMMpass. The APOBEC signature has previously been reported to be associated with a worse patient outcome^21,35^. In this study after adjusting for age, sex, translocation status, chromosome-arm events, and *TP53* status, no statistically significant association was shown, suggesting that APOBEC status does not represent an independent biomarker of patient outcome: progression-free survival (PFS: hazard ratio [HR] = 2.45, 95% confidence interval [CI] = 0.94–6.37, *P* *=* 0.066) and overall survival (OS: HR = 2.81, 95% CI = 0.96–10.10, *P* *=* 0.10) (Supplementary Table 19). We next explored whether incorporating information on major SNVs and SV mutational signatures could further enhance the prediction of patient outcome after taking into account of established prognostic factors. Unsupervised hierarchical clustering provided evidence for seven distinct groups (A–G) associated with both PFS (log-rank *P* = 3.4 × 10^−4^) and OS (log-rank *P* = 0.011) (Fig. 4, Table 2, Supplementary Fig. 8), with group C being enriched for HD MM, group G is featuring tumors with 1p deletion, while group D being characterized by APOBEC mutation, enrichment for *MAF*-translocation subgroups, 1p deletion, and 1q gain (Supplementary Table 20). Post hoc delineation allowed us to stratify patients in seven groups into low- (A, B, C, and E) and high-risk groups (D, G, and F) (Supplementary Table 21). Classification of MM based on mutational signatures captured by these seven groups is independent prognosis factors (Supplementary Table 22). Notably, group F was independently associated with adverse prognosis (PFS: HR = 1.95, 95% CI = 1.35–2.81, *P* *=* 3.3 × 10^−4^ ; OS: HR = 1.47, 95% CI = 1.02–2.13, *P* = 0.039) (Supplementary Table 22), despite not being associated with the high-risk features of APOBEC, t(14;16)/t(14;20), 1p/1q/17p chromosome-arm events or *TP53* mutation status, but was typified by non-clustered structural rearrangements (Fig. 5a, Table 2, Supplementary Fig. 8).

**Table 2:** Summary of characteristics of the seven cluster subgroups

Cluster	*n*	SV features	SNV features	Subgroup association	Known prognostic events
A	155	Clustered translocations		Enriched for t(11;14) and t(4;14)	*TP53* mutations
B	172	Non-clustered small-scaled deletions and tandem duplications			
C	138	Mixture of non-clustered SVs		Enriched for hyperdiploidy	
D	35	Mixture of non-clustered SVs	APOBEC mutations	Enriched for t(14;16) and t(14;20)	1p deletion and 1q gain
E	99	Non-clustered translocations			
F	97	Mixture of non-clustered SVs			
G	154	Large-scaled non-clustered deletions, tandem duplications, and inversions		Enriched for t(4;14)	1p deletion

*SV* structural variant, *SNV* single-nucleotide variant, *APOBEC* apolipoprotein B editing complex

## Discussion

**Fig. 6:**
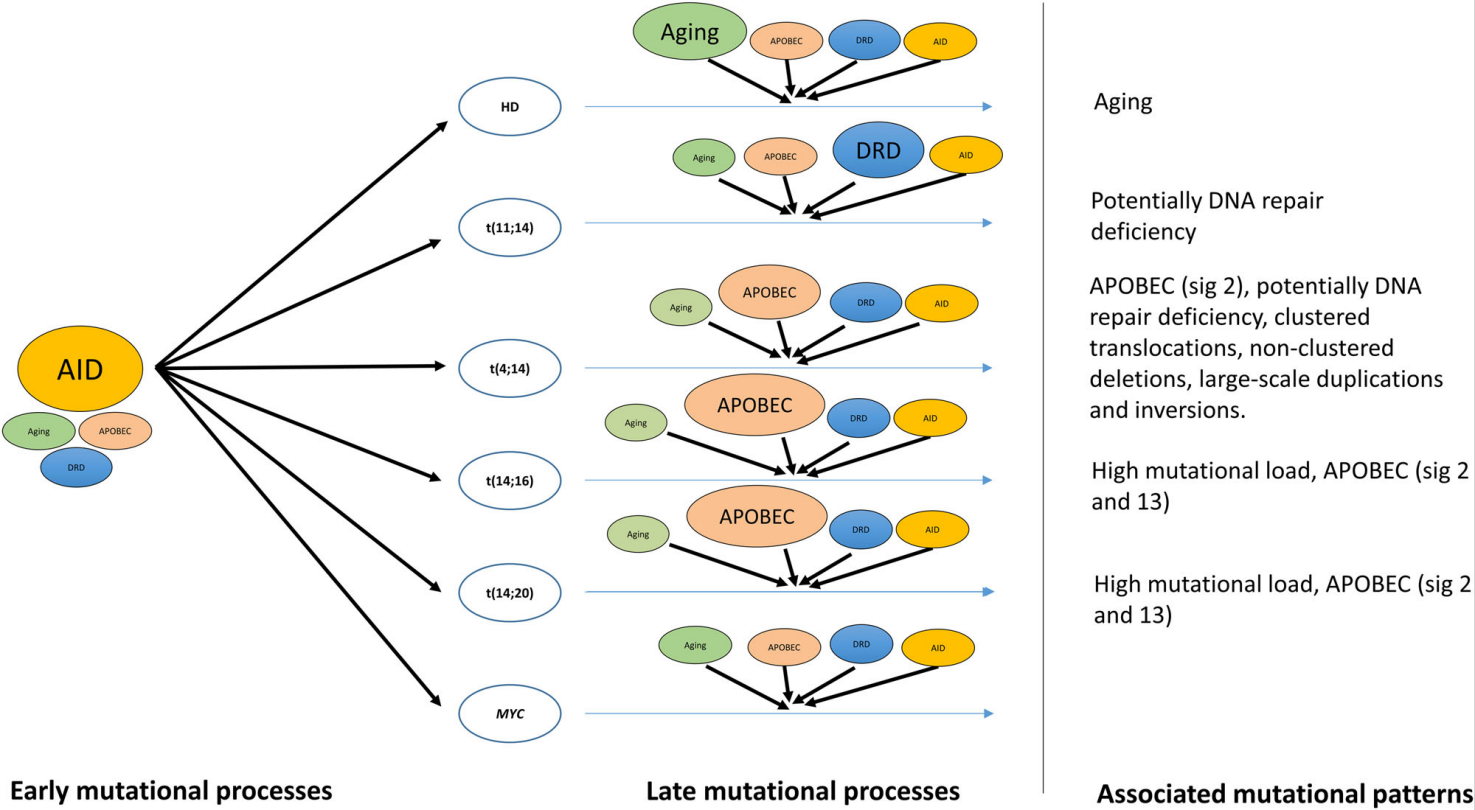
Contribution of major mutational processes operative in multiple myeloma. This model represents differential contribution of various identified mutational processes in myeloma. For early mutational processes, activation-induced deaminase (AID) has the overall largest contribution to mutational processes across all subgroups represented by a larger oval. For late mutational processes, major mutational processes with known etiologies associated with aging, apolipoprotein B editing complex (APOBEC), DNA repair deficiency (DRD), and AID are depicted. Larger oval sizes indicate larger relative contribution of the mutational process

Our analysis of over 800 myeloma genomes has afforded a global overview of the mutational processes in MM tumorigenesis. A major finding of this study is that a combination of signatures linked to aging, APOBEC/AID, and indicative DNA repair deficiency—account for around 80% of mutations in MM. Despite the difficulty of assigning flat signatures (3, 5, and 8)^22,23^, their detection of such profiles in large patient series supports the role of defective DNA repair in MM. By utilizing both WES and WGS data, we were able to extract five novel structural RSs and identify differential prevalent mutational processes in coding (aging and APOBEC) and non-coding regions (AID), consistent with a previous report^30^. Our work supports previous findings^30^ in implying an early role for AID in shaping the MM mutational landscape. We also identified new and validated previously reported subgroup associations with mutational signatures, allowing further categorization of MM beyond simple translocation status and providing additional insight in the etiological processes implicated in tumorigenesis (Fig. 6).

Mutations do not occur uniformly over the genome and local mutation rates are modulated by replication, transcription, and chromatin organization^12^. We observed an enrichment of somatic mutations in late-replicating regions, as seen across several cancers^36^, and highly expressed regions. Previous analyses, which have sought to establish the mutational profile of myeloma genomes, have been based on data solely from exome sequencing projects. Here we have sought to provide a more comprehensive analysis; however, we acknowledge that the low coverage of CoMMpass WGS raises the possibility that we may have underestimated the global mutation rate. The strong replicative asymmetry observed is consistent with mutations in MM being predominantly associated with APOBEC family of mutations^24^. In addition, we identified that coding drivers are likely to be originated from a number of mutational processes, including aging and DNA repair deficiency. In contrast, while APOBEC enzymes appear to act more ubiquitously within coding regions, they do not specifically affect coding drivers.

The different MM translocation subgroups showed striking differences in their mutational signatures, reflective of the cellular processes driving respective clonal expansions (Fig. 6). As previously reported, t(14;16) and t(14;20) MM were enriched with APOBEC signatures 2 and 13^7,35^. This is a consequence of the over-expression of APOBEC genes, specifically *APOBEC3A* and *APOBEC3B*, mediated through the over-expression of MAF transcription factors^35^. The t(4;14) subgroup was also enriched with APOBEC mutational patterns, although only for signature 2 and, to a lesser extent, as compared to *MAF*-translocation subgroups. Since signatures 2 and 13 are reflective of different mutational processes^12^, we speculate that the mutational processes associated with t(4;14) are likely to be different from those with *MAF*-translocation subgroups. In contrast, signatures indicative of DNA repair deficiency were associated with t(11;14) and t(4;14) and aging with HD. DNA breaks unsuccessfully repaired due to defective DNA repair may facilitate the generation of chromosomal translocations^37^. Because of the flat structure of signatures 3, 5, and 8 robust insight into etiological contribution of DNA repair deficiency to MM tumorigenesis requires assiduous signature fitting and adjustment for confounding covariates^23^. The molecular mechanisms responsible for initiating HD in MM are unknown. However, by inference from childhood acute lymphoblastic leukemia^38^, it is likely it is a consequence of the simultaneous gain of chromosomes in a single abnormal cell division. Cells failing to execute programmed cell death in response to mitotic failure are likely to divide asymmetrically, resulting in the generation of aneuploidy cells^39^. The association between aging with increased cell division errors^40^ and decreased apoptosis^41^ further supports a relationship between HD MM and aging. Signatures defined by large-scale structural aberrations were associated to varying degrees with MM subgroups, but clustered translocations and non-clustered deletions, large-scale tandem duplications, and inversions showed a significant association in t(4;14) MM.

The APOBEC mutational signatures are inextricably linked to a high mutation load^7,35^ and the adverse t(14;16) and t(14;20) *MAF*-translocation subgroups. We show that molecular classification based solely on APOBEC signatures do not fully differentiate the underlying genomic complexity in MM relevant to predicting patient outcome. Hence, while APOBEC activity is an adverse prognostic factor in MM^21,35^, using it as a sole classifier does not fully capture high-risk MM, which with genetically unstable genome is typified by complex structural variants. Our findings support the need for considering other mutational signatures to refine prediction of patient prognosis.

Our study does, however, suggest that analysis of APOBEC activity together with other molecular features at diagnosis should allow for the identification of high-risk MM patients that may benefit from more intensive treatment. Collectively, these data shed new light on the diversity of cellular processes generating somatic mutations in MM. Moreover, they provide a strong rationale for integration of mutational signatures data in conventional molecular profiling of patient tumors to tailor therapy.

## Materials and methods

### Samples and datasets

All data analyzed were generated as part of the Multiple Myeloma Research Foundation (MMRF) CoMMpass Study (release IA10). WGS data on 850 matched tumor-normal baseline newly diagnosed bone marrow samples were downloaded from the database of Genotype and Phenotype (dbGaP). Matched tumor RNAseq processed by HTseq were used for gene expression analysis. WES variants (detected by at least two out of three variant callers—MuTect, Seurat, and Strelka) from 874 samples, RNAseq, copy number variation (CNV), clinical data, and sequencing-based fluorescent in situ hybridization (Seq-FISH) data (MMRF IA10 dataset) were downloaded from MMRF web portal (https://research.themmrf.org/). WES and WGS data were available for 824 samples.

### Somatic mutation calling

Calling of somatic mutations was performed as described previously^7^. Briefly, raw WGS sequencing data were quality checked using FastQC (v.0.11.4) and aligned using the Burrows-Wheeler Alignment tool^42^ (BWA v0.7.12) to the human genome hg19/GRCh37 assembly. SNV mutations were called using MuTect^43^ (v1.1.7) according to best practices, utilizing data from dbSNP v147 and COSMIC non-coding variants v77^44^. Mutations were then filtered for oxidation artifacts^45^ and by quality score as described previously^7^. Mutations mapping to immune hypermutated regions (429 immunoglobulin and the major histocompatibility complex loci, each region extended by 50 kb, as defined in Ensembl v73)^46^ were excluded to avoid bias from mutation as a consequence of normal B cell development.

### Determination of myeloma karyotype

Translocation status of MM tumors was based on Seq-FISH^47^. HD was defined as amplification of 90% of the chromosome in at least two autosomes^7^. Prognostic chromosome-arm events (>1 Mb) were defined as deleted or amplified with abs(log_2_ ratio) ≥0.1613 occurring at 1p12, 1p32.3, 1q21.1, 1q23.3, and 17p13^6^.

### Mutational signatures

Characterization of the 30 COSMIC mutational signatures (http://cancer.sanger.ac.uk/cosmic/signatures) and de novo extraction of signatures was performed using Palimpsest^11,48^ with default parameters. We compared de novo mutational signatures with 30 pre-defined COSMIC signatures by computing their cosine similarities^1^. A de novo mutational signature was assigned to a COSMIC signature if the cosine similarity was >0.75 as previously advocated^11^. If multiple COSMIC signatures passed this threshold, then the most similar COSMIC signature was assigned to the de novo signature. We compared proportion COSMIC mutational signatures between high-coverage WES clonal mutations (alternate allele ratio >0.9) and low-coverage WGS mutations restricted to exome regions, as well as between CoMMpass exome and Walker et al.^8^ exome mutations. Correlations were tested using Spearman’s correlation. For those signatures with an apparent flat profile we considered these in concert, by combining the respective contributions of signatures 3, 5, and 8.

We used MANTA to identify somatic SVs from the WGS data adopting default settings^49^. We applied the same statistical framework used for signature analysis of SVs implemented in Palimpsest^48^ to extract de novo RSs as previously described^11^. Correlations between SV signatures and major COSMIC pre-defined SNV signatures (>1% mutational contribution in WGS) were tested using Spearman’s correlation. No significant correlation was seen after adjusting for multiple testing (i.e., *Q* > 0.05).

We examined the relationship between mutational signatures and clinico-pathological parameters confining our analysis to the major MM subgroups—HD, t(4;14), t(11;14), t(14;16), t(14;20), and t(8;14) *MYC*. Test of association between each signature and subgroups was based on a two-tailed Fisher’s exact test using Benjamini–Hochberg false discovery rate procedure to address multiple testing.

We compared contribution of each mutational signature to coding and non-coding regions using WGS data. To calculate contribution of a mutational signature to a genomic region, we first estimated the probability that each mutation was due to the process underlying each signature and calculated the cumulative probability of all mutations in each region, as per Letouze et al.^11^. After computing these probabilities, regional differences in trinucleotide composition were accounted for when comparing the contribution of mutational signatures between two genomic regions (regions *X* and *Y*). Such normalization was conducted by changing the number of mutations from each mutational category in region *X* to that expected if the trinucleotide composition of region *X* was identical to the trinucleotide composition of region *Y*, assuming a constant rate of mutation at positions of each trinucleotide context. The normalized number of mutations $U_{\mathrm{norm}}^{C,X}$ of category *C* in region *X* was calculated as:

$$U_{\mathrm{norm}}^{{C},{X}} = U^{C,X}\frac{{V^{C,Y}{W}^X}}{{V^{{C},{X}}{W}^{Y}}},$$

where *U*^*C,X*^ is the number of mutations of category *C* observed in region *X*, *V*^*C,X*^ is the number of positions at which a mutation of category *C* can occur in region *X*, and *W*^*X*^ is the size of region *X* (in base pairs). As $U_{\mathrm{norm}}^{C,X}$ is not necessarily an integer, it is rounded to the closest integer before comparisons are completed. Mutation numbers were normalized within each tumor. Since small numbers of mutations may impact on normalization, in each comparison the larger region was designated as region *X*, the smaller region designated as region *Y*.

### Replication timing and replication strand bias

We used replication sequencing (Repli-seq) data generated by the ENCODE consortium for the lymphoblast cell lines GM12878, GM06990, GM12801, GM12812, and GM12813 to define early- and late-replicating regions, as well as leading and lagging DNA strands using Repli-seq signal peaks from GM12801 as previously described^11,12^. Mutation rates across deciles of replication timings were estimated globally using WGS data and for each signature, with each mutation assigned to a single signature by Palimpsest^11,48^. The replication timing slope was estimated by linear regression model. To test the null hypothesis that the slope gradients equal zero, the replication timing deciles were permuted 10,000 times. Empirical *P* values were calculated as the fraction of permutations with absolute slope values at least as great as the absolute slope value computed using the true replication timing deciles.

Analysis of mutational replication strand bias between leading and lagging strands was performed across all 30 COSMIC signatures as previously described^11^, using WGS data. The Wilcoxon’s rank-sum test was used to determine significant difference of mutational contribution from each COSMIC signature between leading and lagging strands. Levels of asymmetry were considered significant if strand imbalances were >30%^12^ and *Q* < 0.05.

### Transcriptional levels and transcriptional strand bias

To correlate mutational processes with gene expression, RNAseq data were normalized to FPKM^11^. For each tumor, genes were partitioned into pentiles based on respective FPKM. Immunoglobulin-related genes and genes known to be highly upregulated in MM as a result of translocations (*CCND1*, *CCND3*, *FGFR3*, *MMSET*, *MAF*, *MAFB*, and *MYC*)^6^ were excluded to mitigate against bias. Mutation rates of genes within each of the five transcriptional level categories were estimated per tumor based on WES called mutations. Average alignability score for highly expressed genes was based on alignability of 75mers defined by the ENCODE/CRG GEM mappability tool^50^. We examined mutation rates on transcribed and non-transcribed strands globally and for each signature as described previously^11^ using Palimpsest^11,48^. Wilcoxon’s rank-sum tests, corrected for multiple testing, were used to determine significant difference of mutational contribution from each COSMIC signature between transcribed and non-transcribed strands. Levels of asymmetry were again considered significant if strand imbalances were >30%^12^ and *Q* < 0.05.

### Kataegis

We restricted our kataegis analysis to high-coverage WES data, where we have sufficient coverage to detect local hypermutation. Kataegis foci were defined as having six or more consecutive mutations with an average mutational distance ≤1 kb, as previously described^3,32^. Co-localization of kataegis and structural rearrangements was assessed based on the proportion of SV regions having kataegis foci residing within 10 kb. To examine enrichment of a mutational signature at kataegis regions, we compared mutational contribution of each signature across all mutations at kataegis foci with other mutations in tumors with and without kataegis being detected using Wilcoxon’s rank-sum test, corrected for multiple testing and imposed a threshold of *Q* < 0.05.

### Association of mutational signatures with the mutation of driver genes

For SNV mutational signatures, Wilcoxon’s rank-sum tests were used to compare contribution of each mutational signature in coding drivers^7–10^ and other exonic mutations, with normalizing for trinucleotide composition as described above. For each somatic mutation, we estimated the probability that it was the result of each mutational process considering the trinucleotide context and the number of mutations attributed to each process in the respective tumor as per Letouze et al.^11^. We then compared, for each driver gene and mutational signature, the probability distribution in mutations affecting the driver gene as compared to all other mutations in tumors with and without the driver gene mutated using Wilcoxon’s rank-sum tests, imposing Benjamini–Hochberg correction for multiple testing. We evaluated all driver genes identified in previous studies^7–10^ with *Q* < 0.05.

### Association of signatures with clinical features

Multivariate Cox regression was performed to adjust for covariates, including age at diagnosis, sex, translocation status, and APOBEC mutational contribution (COSMIC signatures 2 and 13). We used the ConsensusClusterPlus R package^51^ to hierarchically cluster patients based on de novo SV and major COSMIC SNV signatures (>1% contribution) extracted from WGS with default settings^32^. Fisher’s exact test was used to test whether clusters were associated with MM subgroups or driver gene mutations, imposing Benjamini–Hochberg correction for multiple testing. The log-rank test was used to assess the differences in PFS and OS between all cluster groups. To delineate clusters into low- and high-risk groups, pairwise comparisons in survival distributions were performed using the pairwise_survdiff function implemented in the survminer R package^33^.

Multivariate Cox regression was performed for each subgroup vs. other subgroups, adjusting for age at diagnosis, sex, translocation status, APOBEC contribution, 1p deletion, 1q gain, 17p deletion, and *TP53* non-synonymous mutations.

## Supplementary information

Supplementary Figures

Supplementary Tables

## Data Availability

WGS and WES raw fastq data were obtained from dbGaP under the study accession code phs000748.v4.p3. WES somatic variants, RNAseq, CNV, Seq-FISH, and clinical data were obtained from MMRF IA10 (https://research.themmrf.org/). Replication timing data were downloaded from the UCSC Genome Browser (http://hgdownload.cse.ucsc.edu/goldenPath/hg19/encodeDCC/wgEncodeUwRepliSeq/).
