# Supplementary material for: Mutational processes contributing to the development of multiple myeloma
Source: Blood Cancer J. 2019 Aug 6;9(8):60. doi: 10.1038/s41408-019-0221-9 (PMC6684612; doi:10.1038/s41408-019-0221-9)
Supplement: Supplementary file 1 — Supplementary Figures [file 41408_2019_221_MOESM1_ESM.pdf]

Supplementary Figures

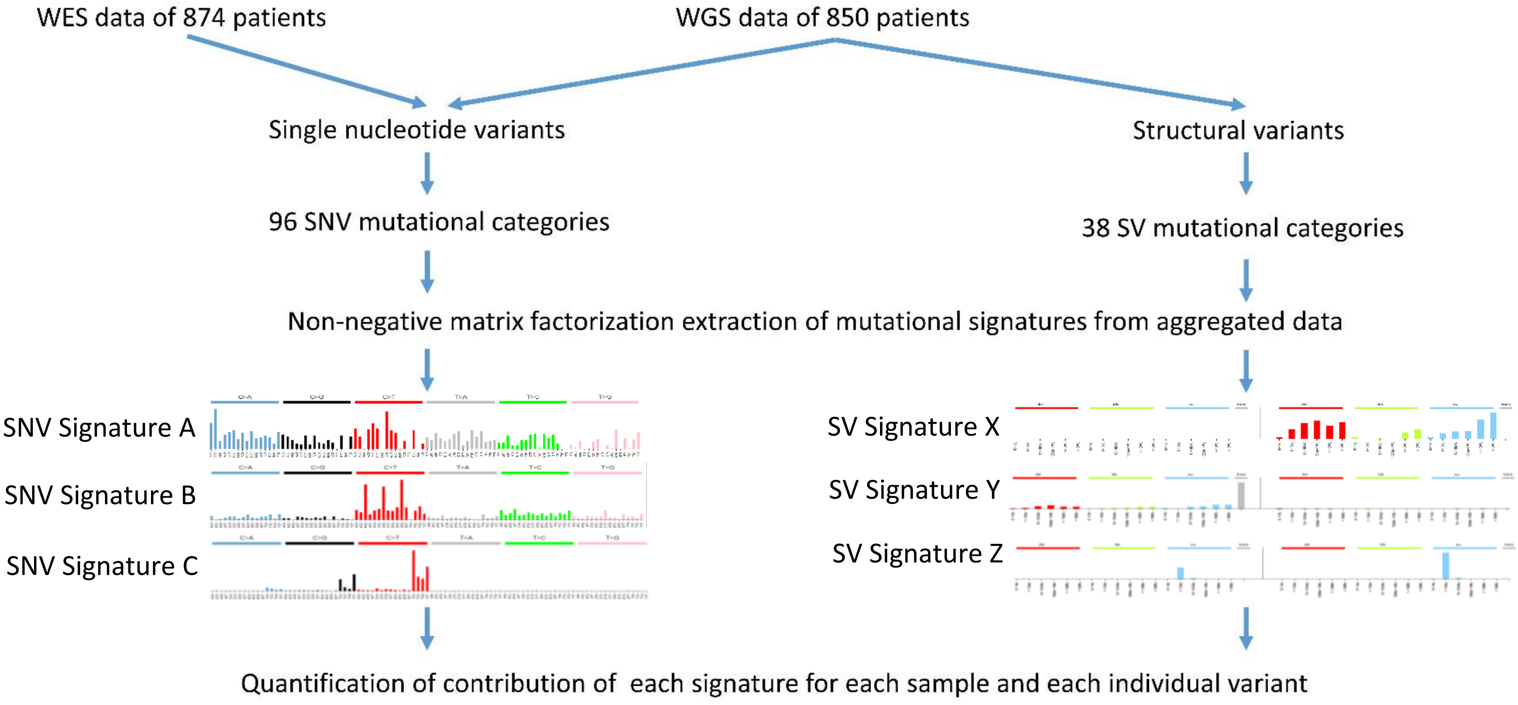

Supplementary Figure 1: **Summary of mutational signatures extraction in the study.** WES: Whole-exome sequencing. WGS: Whole-genome sequencing. SNV: single nucleotide variants. SV: structural variants. Figure adapted from Helleday *et al*[1].

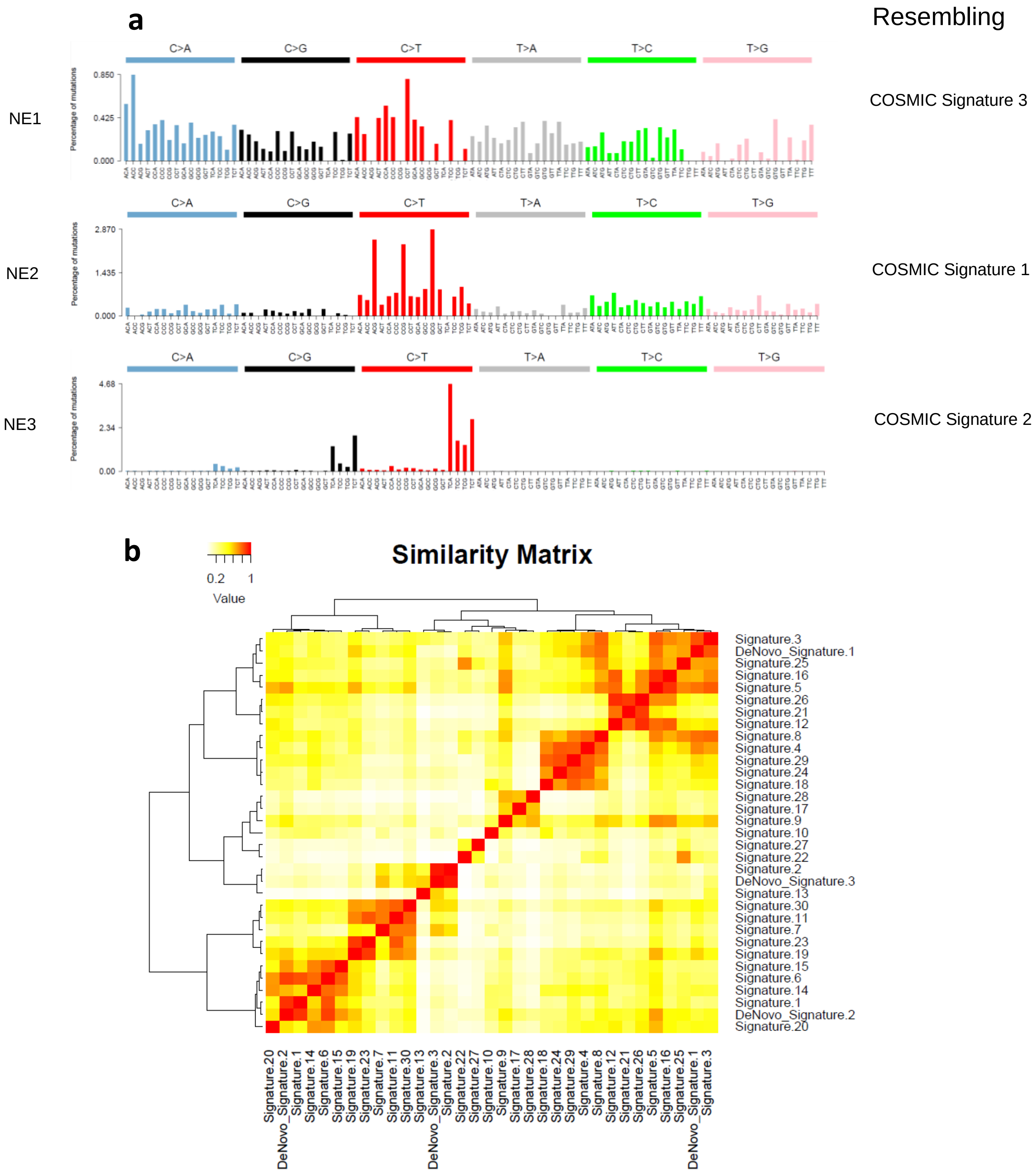

Supplementary Figure 2: ***De novo* extraction of WES single nucleotide variants signatures using non-negative matrix factorization algorithm. (a)** Summary of three *de novo* mutational signatures extracted. **(b)** Cosine similarity heatmap. *De novo* extracted mutational signatures are compared against 30 COSMIC mutational signatures. The colour code (0 to 1) represents the resemblance between each pair of signatures. Signatures are grouped together by hierarchical clustering. Figures are generated using Palimpsest R package. NE: *de novo* exome signature.

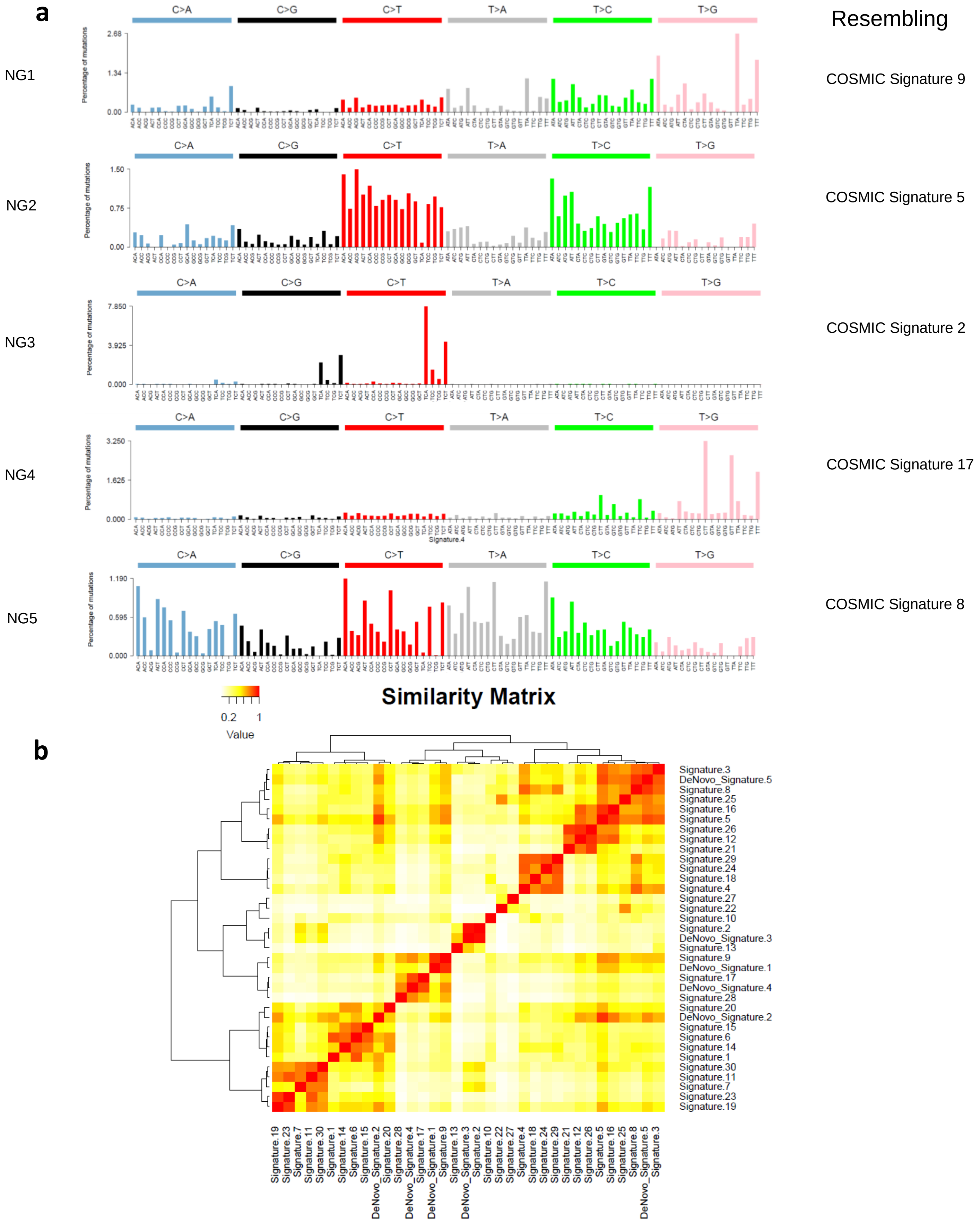

Supplementary Figure 3: ***De novo* extraction of WGS single nucleotide variants signatures using non-negative matrix factorization algorithm. (a)** Summary of three *de novo* mutational signatures extracted. **(b)** Cosine similarity heatmap. *De novo* extracted mutational signatures are compared against 30 COSMIC mutational signatures. The colour code (0 to 1) represents the resemblance between each pair of signatures. Signatures are grouped together by hierarchical clustering. Figures are generated using Palimpsest R package. NG: *de novo* whole-genome signature.

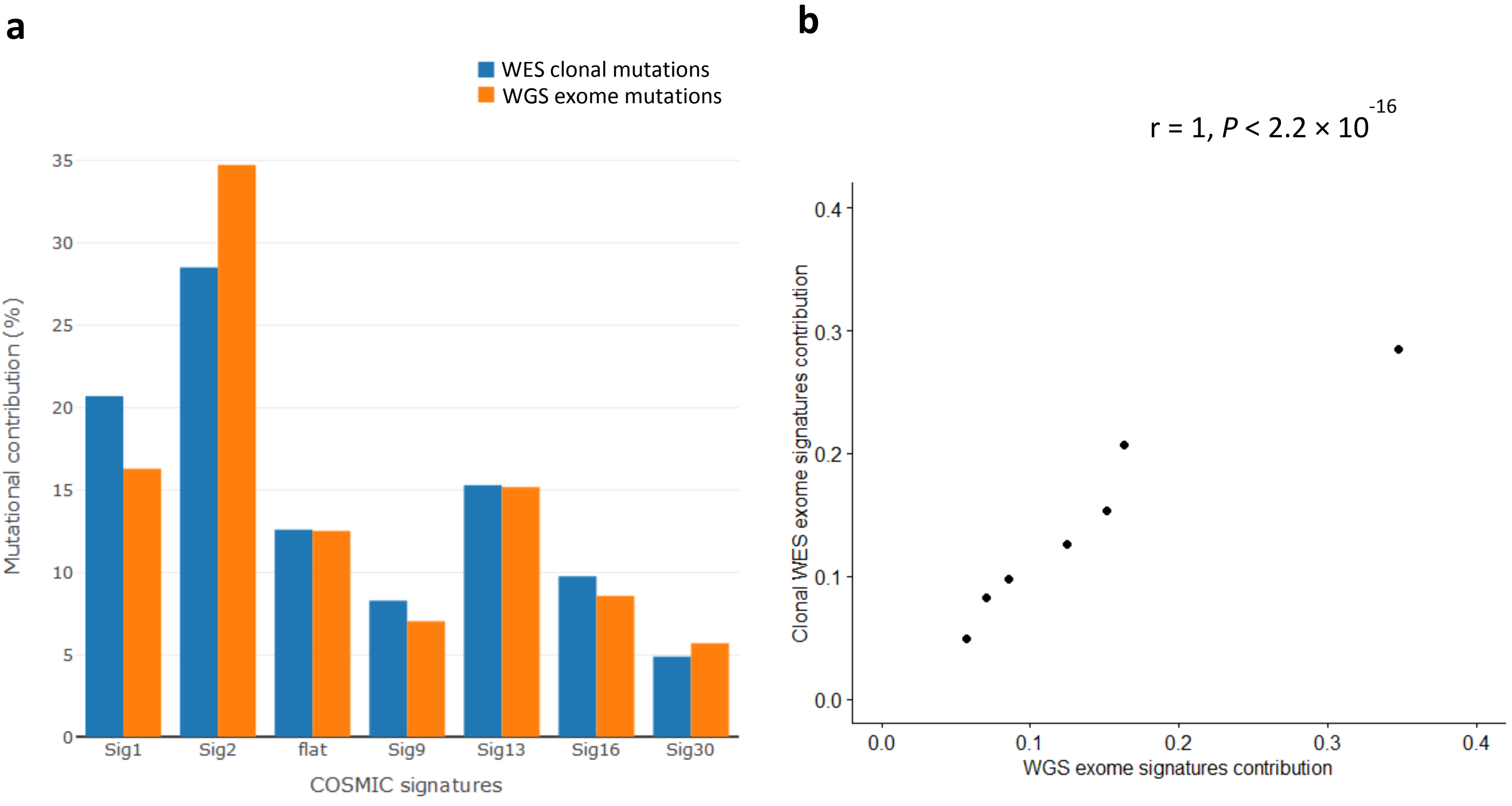

Supplementary Figure 4: **Concordance between clonal whole-exome and exome-restricted whole-genome single nucleotide variants mutational signatures (n = 525). (a)** Cumulative mutational contributions of major COSMIC mutational signatures in clonal whole-exome sequencing (WES, blue) mutations and exome-restricted whole-genome sequencing (WGS, orange). **(b)** Scatter plot showing high concordance (Spearman's correlation) between mutational signatures identified in clonal WES mutations and exome-restricted WGS. Flat signatures include COSMIC signatures 3, 5, and 8.

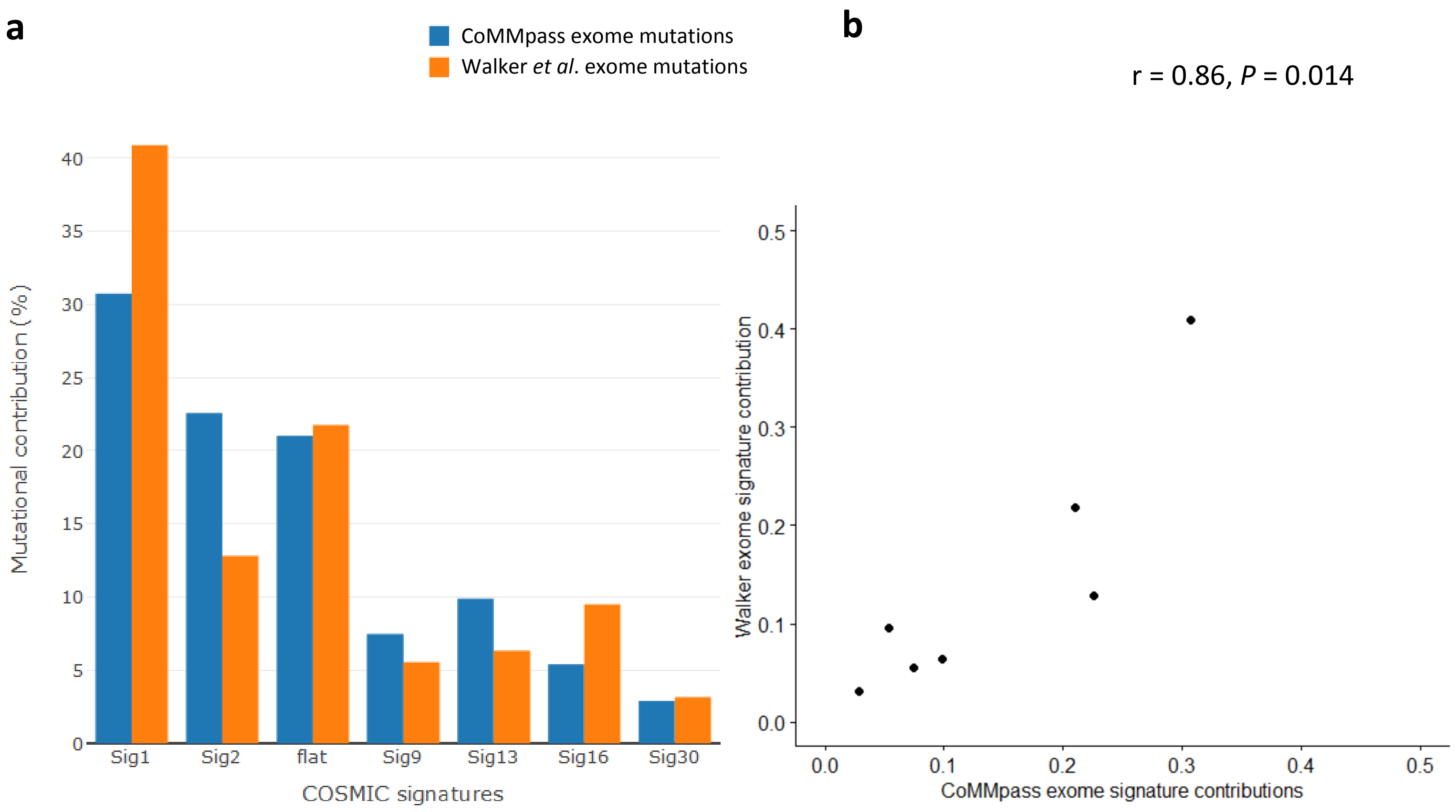

Supplementary Figure 5: **Concordance between CoMMpass and Walker *et al.*[2] exome single nucleotide variants mutational signatures. (a)** Cumulative mutational contributions of major COSMIC mutational signatures in exome variants from CoMMpass (blue, n = 874) and Walker *et al.* exome study (orange, n = 463); and **(b)** Scatter plot showing high concordance (Spearman's correlation) between mutational signatures identified in CoMMpass and Walker's exome mutations. Flat signatures include COSMIC signatures 3, 5, and 8.

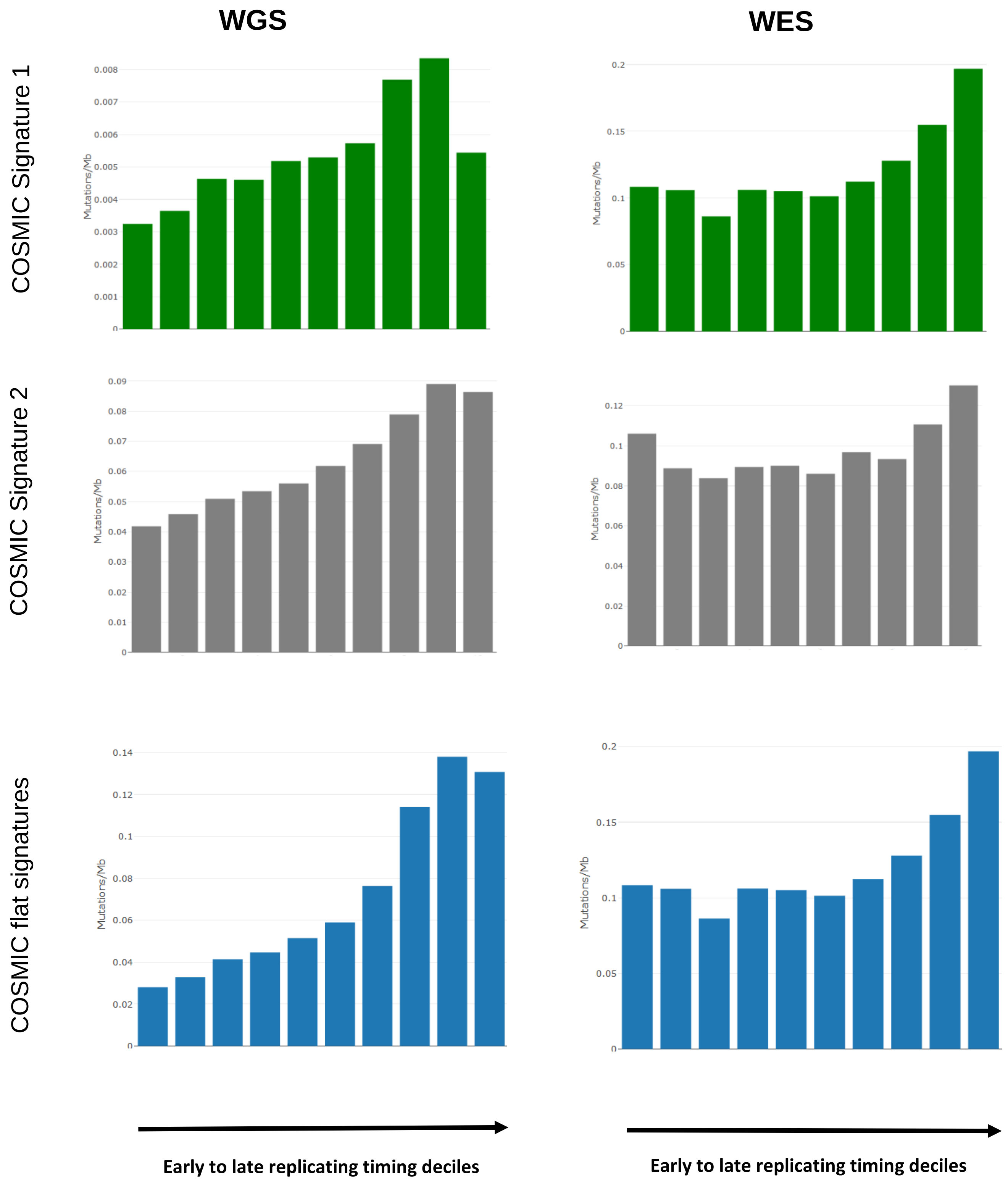
WGS
WES
COSMIC Signature 1
COSMIC Signature 2
COSMIC flat signatures
Mutations/Mb
0
0.001
0.002
0.003
0.004
0.005
0.006
0.007
0.008
0.05
0.1
0.15
0.2
0.01
0.02
0.03
0.04
0.06
0.07
0.08
0.09
0.12
0.14
Early to late replicating timing deciles
Early to late replicating timing deciles

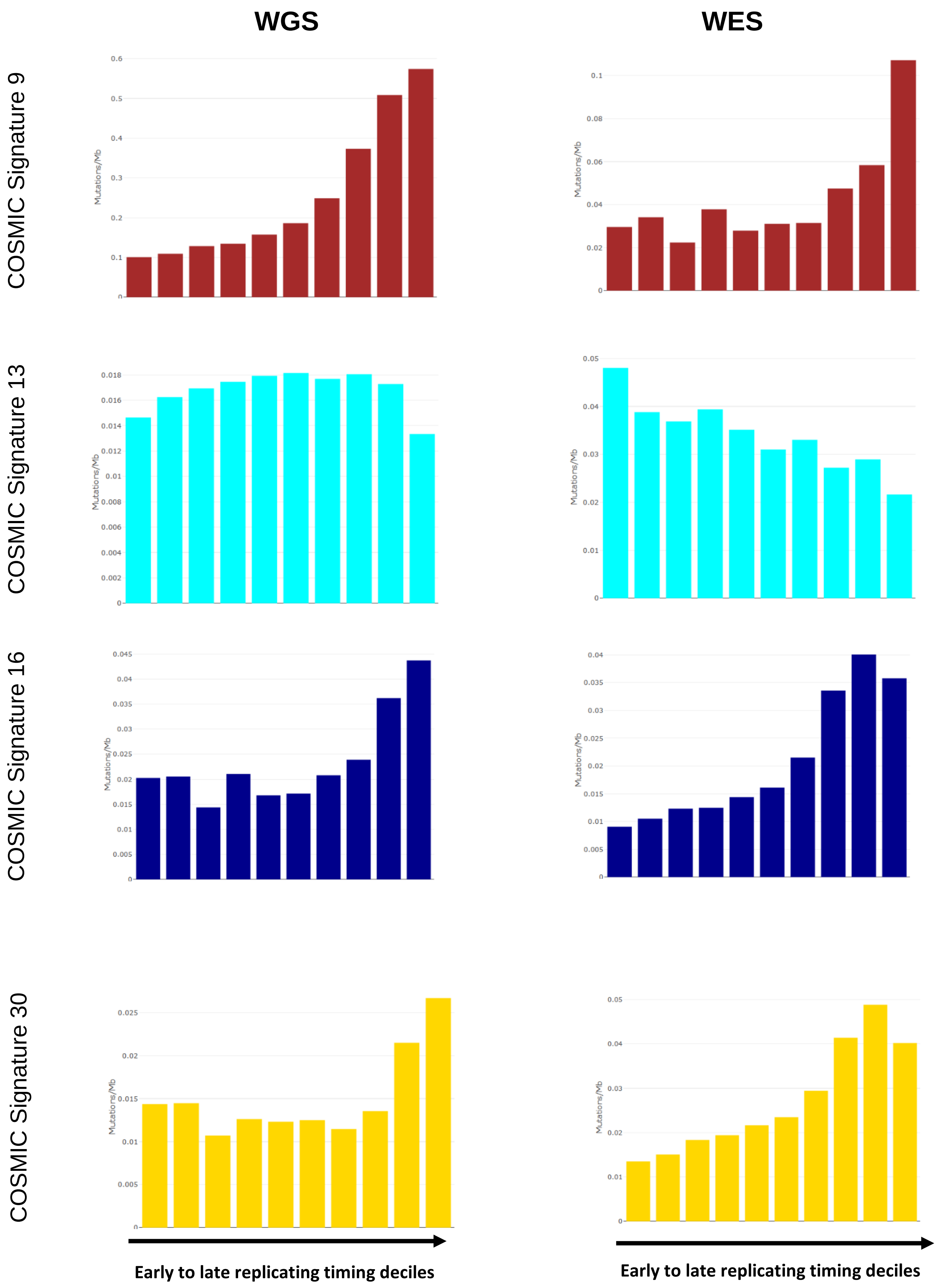
WGS
WES
COSMIC Signature 9
COSMIC Signature 13
COSMIC Signature 16
COSMIC Signature 30
Mutations/Mb
Early to late replicating timing deciles
Early to late replicating timing deciles

Supplementary Figure 6: **Correlation between DNA replication timing and SNV mutation rates per major COSMIC signatures (>1% mutational contribution in the WGS data).** WGS: whole-genome sequencing. WES: whole-exome sequencing. Flat signatures include COSMIC signatures 3, 5, and 8

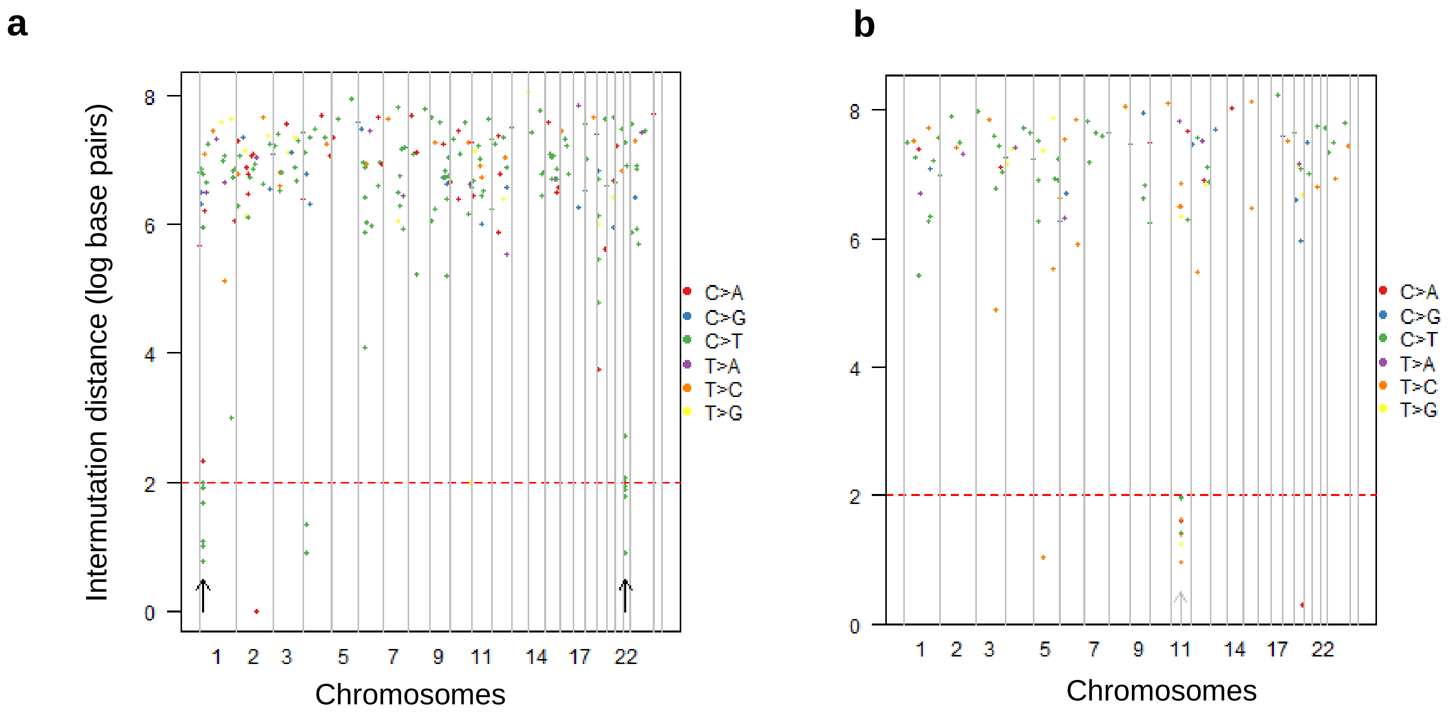

Supplementary Figure 7: **Kataegis plots** (a) MMRF 1579: the kataegis focus on chromosome 1 and 22 detected co-localize with del 1p and an inversion on chromosome 12 respectively; (b) MMRF 2186: the kataegis foci on chromosome 11 co-localizes with t(11;14) (q13;q32). Plots were made using Kataegis Portal R package.

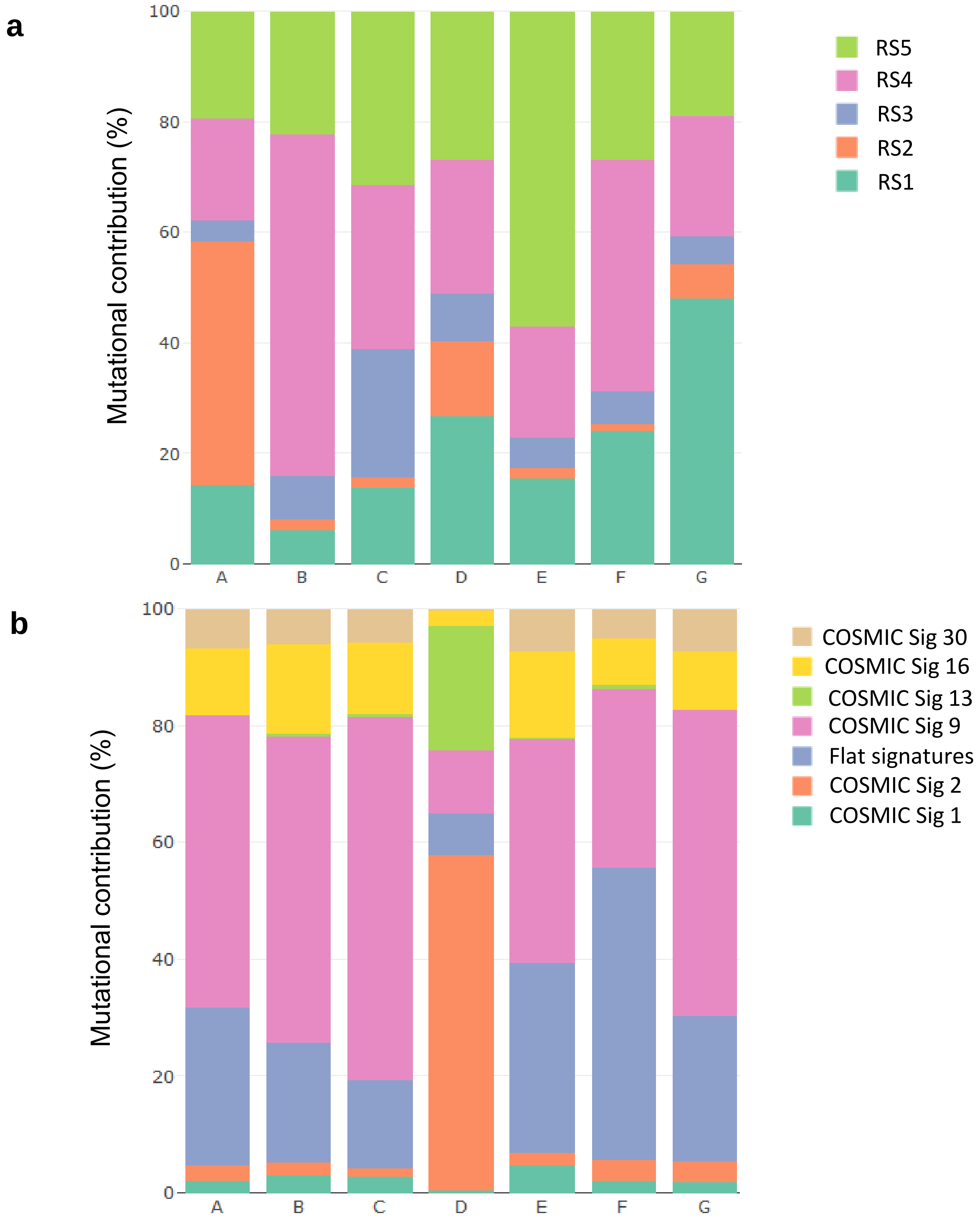

Supplementary Figure 8: **(a)** Structural rearrangements and **(b)** COSMIC single nucleotide variant signatures (>1% contribution across all subgroups) mutational contribution in each of the unsupervised hierarchical clustered subgroups (A – G). RS: structural rearrangement signatures. Sig: signatures.

References

1. Helleday T, Eshtad S, Nik-Zainal S. Mechanisms underlying mutational signatures in human cancers. *Nat Rev Genet* 2014 Sep; **15**(9)**:** 585-598.

2. Walker BA, Boyle EM, Wardell CP, Murison A, Begum DB, Dahir NM*, et al.* Mutational Spectrum, Copy Number Changes, and Outcome: Results of a Sequencing Study of Patients With Newly Diagnosed Myeloma. *J Clin Oncol* 2015 Nov 20; **33**(33)**:** 3911-3920.
